# Effects of cranberry extracts on gene expression in THP‐1 cells

**DOI:** 10.1002/fsn3.374

**Published:** 2016-04-25

**Authors:** Daniel B. Hannon, Jerry T. Thompson, Christina Khoo, Vijaya Juturu, John P. Vanden Heuvel

**Affiliations:** ^1^ Department of Veterinary and Biomedical Sciences and Center for Molecular Toxicology and CarcinogenesisPenn State University325 Life Sciences BuildingUniversity ParkPennsylvania16802; ^2^ Ocean Spray Cranberries, Inc.One Ocean Spray DriveLakeville‐MiddleboroMassachusetts02349; ^3^ UnitedBio‐Med Inc.102 Hunters RunDobbs FerryNew York10502; ^4^ INDIGO Biosciences Inc.1981 Pine Hall RoadState CollegePennsylvania16801

**Keywords:** Gene expression, macrophage, nutrigenomics, proanthocyanidins, reactive oxygen species

## Abstract

Cranberry contains high levels of nutrients and bioactive molecules that have health‐promoting properties. The purpose of the present studies was to determine if cranberry extracts (CEs) contain phytochemicals that exert anti‐inflammatory effects. The human monocytic cell line THP‐1 was treated with two CEs (CE and 90MX) and subsequently challenged with Lipopolysaccharides (LPS). Tumor necrosis factor *α* (TNF *α*) expression was decreased in the CE‐treated cells, indicative of an anti‐inflammatory effect. Gene expression microarrays identified several immune‐related genes that were responsive to CEs including interferon‐induced protein with tetratricopeptide repeats 1 and 3 (IFIT 1 and 3), macrophage scavenger receptor 1 (MSR1) and colony‐stimulating factor 2 (CSF2). In addition, in the CE‐treated cells, metallothionein 1F and other metal‐responsive genes were induced. Taken together, this data indicates that CEs contain bioactive components that have anti‐inflammatory effects and may protect cells from oxidative damage.

## Introduction

Epidemiological, human intervention, and mechanistic trials have shown that polyphenol‐rich foods, often found in a diet rich in fruits and vegetables, may have protective effects against chronic degenerative diseases linked to oxidative stress and reactive oxygen species (ROS)‐mediated cell damage (Cassidy et al. 2011; Wedick et al. 2012; Del Rio et al. 2013). Flavonoids and other phenolic compounds in these foods are bioactive health components that are likely responsible for these effects. The North American cranberry (*Vaccinium macrocarpon* Ait. Ericaceae) is of growing public interest as a functional food because of potential health benefits linked to the high content of bioactive flavonoids in the fruit, including proanthocyanidins, flavonols such as quercetin and myricetin and anthocyanins. In fact, cranberry ranks high among fruit in both antioxidant quality and quantity (Vinson et al. 2001; Gu et al. 2004; Côté et al. 2010), and contain a higher amount of total phenols per serving (507–709 mg gallic acid equivalents/100 g) than other common fruits including blueberries (258–531 mg/100 g), apples (185–347 mg/100 g), and red grapes (175–370 mg/100 g) (Wu et al. 2004). Cranberries, in powder, extract or juice, have been reported to be beneficial for helping prevent urinary tract infections (Wang et al. 2012; Blumberg et al. 2013; Foxman et al. 2015), oxidative stress and cardiometabolic risk factors such as reducing C‐reactive protein and lowering blood pressure (Basu et al. 2011; Dohadwala et al. 2011; Novotny et al. 2015). As cranberry is a natural food product and not a drug, the effect seen is often mild but that is consistent with the recommendation for a food with health components that is consumed as part of a healthy balanced lifestyle.

In vitro evidence has shown the antioxidant potential of cranberry phenolic compounds including protection of human microvascular endothelial cells against ROS, and HepG2 cells from inflammatory insults and reduced glycation (Caton et al. 2010; Crozier et al. 2010; Liu et al. 2011; Watson et al. 2014; Martín et al. 2015). A recent publication also showed the bioavailability and bioactivity of cranberry phenolics and metabolites from consuming cranberry juice beverage (McKay et al. 2015). The protective effect of these compounds may be related to their function in sequestering ROS and/or maintaining the cell components in their correct redox state, but emerging findings indicate that natural compounds may act by increasing the endogenous antioxidant defense potential through regulation of cell signaling pathways (Seeram et al. 2004; Stevenson and Hurst 2007; Martín et al. 2015). The objective of this study is to examine the anti‐inflammatory activity of cranberry phenolic compounds in two different cranberry powders (90 MX and CE) by looking at changes in gene expression using human monocytic cells lines.

## Material and Methods

### Chemicals

Cranberry juice powder and extract were obtained from Ocean Spray Cranberries (Lakeville‐Middleboro, MA; for chemical analysis see Table 1 and reference [Martín et al. 2015]). Cranberry juice powder (90MX) was prepared from the juice of mature berries of the commonly cultivated cranberry plant (*Vaccinium macrocarpon*). Cranberry juice processing consists of the milling and pressing of the berries after a hot (50°C for 1 h) commercial pectinase maceration. 90MX was then prepared by spray drying cranberry concentrate with magnesium hydroxide as the carrier and tri‐calcium phosphate as an anti‐caking agent. The powder is fine, free‐flowing and rosy red in color, and contains approximately 90% cranberry solids. The cranberry extract (CE) powder is a water‐soluble, phenolic‐rich extract of cranberry utilizing a proprietary resin separation process. CE was standardized to 55% proanthocyanidin (PAC) content on a dry weight basis as analyzed by a modified 4‐dimethylaminocinnamaldehyde (DMAC) method (Martín et al. 2015). Finally, 100 mg/mL stock solutions of 90MX and CE were prepared in Dimethylsulfoxide (DMSO) for cell treatment. All media components and fetal bovine serum (FBS) were purchased from Gibco BRL/Life Technologies (Carlsbad, CA). Phorbol 12‐myristate 13‐acetate (PMA), used to differentiate THP‐1 cells, was purchased from Sigma Chemical Company (St. Louis, MO). All other chemicals and reagents were of highest quality available.

**Table 1:** Content of phenolic compounds and other major components in cranberry juice and extract powders

	Cranberry juice powder (90MX, mg/100 g)	Cranberry extract powder (CE, mg/100 g)	Enrichment (CE/90MX)^c^
Proanthocyanidins			
By OS‐DMAC method^a^	2800	51,600	18.4
By BL‐DMAC method^b^	653	15,000	23.0
Organic acids			
Citric acid	14,200	2850	0.2
Malic acid	8510	1660	0.2
Quinic acid	13,500	2040	0.2
Galacturonic acid	3300	78.4	0.0
Sugars			
Glucose	31,300	4700	0.2
Fructose	8120	1100	0.1
Anthocyanins			
Cyanidin‐3‐arabinoside	33.8	420	12.4
Cyanidin‐3‐galacoside	41.7	508	12.2
Cyanidin‐3‐glucose	1.3	25.4	19.5
Peonidin‐3‐arabinoside	17.3	239	13.8
Peonidin‐3‐galactose	41.9	530	12.6
Peonidin‐3‐glucoside	3.0	36.6	12.2
Phenolic acids			
Benzoic acid	84.5	2550	30.2
Salicylic acid	0.6	13.4	22.3
Protocatechuic acid	15.6	120	7.7
Gallic acid	4.2	25.8	6.1
Vanilic acid	4.9	85.4	17.4
*t*‐Cinnamic acid	7.7	72.8	9.5
*p*‐Coumaric acid (4‐hydroxycinnamic acid)	30.2	436	14.4
Caffeic acid (3,4‐dihydroxycinnamic acid)	3.0	50.5	16.8
Ferulic acid (3‐methoxy‐4‐hydroxycinnamic acid	0.9	54.7	60.8
Chlorogenic acid	22.8	793	34.8
Ellagic acid	1.3	2.2	1.7
Flavon‐3‐ols			
Catechin	8.0	43.3	5.4
Epicatechin	31.7	2.2	0.1
Flavonols			
Quercetin	37.3	404	10.8
Quercitrin (quercetin‐3‐*O*‐rhamnoside	20.8	527	25.3
Hyperoside (quercitin‐3‐*O*‐galactoside)	53.1	2720	51.2
Myricetin	40.5	657	16.2
Myricetrin (myrocetin‐3‐*O*‐rhamnoside)	7.6	125	16.4
Total phenolic by Folin‐Ciocalteu method (GAE)	2600	45,000	17.3

From Martín et al. (2015).

a Expressed in milligram (mg) of cranberry‐specific PACs equivalents per 100 g of powder.

b Expressed in milligram (mg) of procyanidine A‐2 equivalents per 100 g of powder.

c Enrichment is the ratio of each component in the CE extract relative to that of 90MX.

### Cell culture and treatment

THP‐1 (*Homo sapiens* monocyte) cells were obtained from the American Type Culture Collection (ATCC; Rockville, MD) and cultured in RPMI 1640 with 10% heat inactivated FBS, 50 *μ*mol/L 2‐mercaptoethanol, 1 mmol/L sodium pyruvate, 100 U/mL penicillin and 100 *μ*g/mL streptomycin. These cells were seeded in 24‐well plates at a density of 3 × 10^5^/well and differentiated into macrophages with 100 nmol/L PMA (Sigma) for 48 h. For in vitro treatment experiments, THP‐1 cells were grown to 75% confluency and treated with 90MX or CE (0, 25, 50, 100 *μ*g dry weight/mL media, heretofore listed as *μ*g/mL). Sixteen hrs after treatment, the cells will be stimulated with 10 ng/mL LPS for 6 h.

### RNA extraction, reverse transcription, real‐time PCR

Total RNA was isolated by Qiagen RNeasy Mini Kit (Qiagen, Valencia, CA) according to the manufacturer's instructions. The total RNA was reverse transcribed, using the ABI High Capacity cDNA archive kit (Applied Biosystems, Foster City, CA). Standard curves were made using serial dilutions from pooled cDNA samples. Real‐time polymerase chain reaction (PCR) was performed with the use of the SYBR Green PCR Master Mix (Applied Biosystems) according to the manufacturer's protocol and amplified on the ABI Prism 7000 Sequence Detection System. Primers are listed in Table 2.

**Table 2:** Primer sequences used for quantitative real‐time PCR

Gene	Forward primer	Reverse primer
18S	ACCCGTTGAACCCCATTCGTGA	GCCTCACTAAACCATCCAATCGG
ABCG2	ACGAACGGATTAACAGGGTCA	CTCCAGACACACCACGGAT
CAT	TGTTGCTGGAGAATCGGGTTC	TCCCAGTTACCATCTTCTGTGTA
CCNL2	GTACTCCGGGGTGCTCATC	GAGGTCGGTCTCTGTGTCG
COX2	CGGTGTTGAGCAGTTTTCTCC	AAGTGCGATTGTACCCGGAC
CSF2	TCCTGAACCTGAGTAGAGACAC	TGCTGCTTGTAGTGGCTGG
HERC5	GGTGAGCTTTTTGCCTGGG	TTCTCCGGCAGAAATCTGAGC
IFIT1	GCGCTGGGTATGCGATCTC	CAGCCTGCCTTAGGGGAAG
IFIT3	AGAAAAGGTGACCTAGACAAAGC	CCTTGTAGCAGCACCCAATCT
IL‐1*α*	CTCCCAATCTCCATTCCCAA	CGTAAGGCCTCAGCCAGAAG
IL6	GCCACTCACCTCTTCAGAACG	CCGTCGAGGATGTACCGAATT
MSR1	GCAGTGGGATCACTTTCACAA	AGCTGTCATTGAGCGAGCATC
MT1F	TCCTGCAAGAAGAGCTGCTG	ACTTCTCTGACGCCCCTTTG
OAS1	TGTCCAAGGTGGTAAAGGGTG	CCGGCGATTTAACTGATCCTG
SLC7A11	TCTCCAAAGGAGGTTACCTGC	AGACTCCCCTCAGTAAAGTGAC
SOD1	GGTGTGGCCGATGTGTCTAT	CCTTTGCCCAAGTCATCTGC
TNFA	ATCAATCGGCCCGACTATCTC	TGGATGTTCGTCCTCCTCACA

### Microarray experiments and statistical analysis

THP‐1 cells were treated with CE or 90MX at 100 *μ*g/mL or control (DMSO, 0.1% v/v) for 16 h followed by stimulation with LPS for 6 h. RNA was extracted, using Qiagen RNeasy and quality assessed by RNA Nano Chips on the Agilent Bioanalyzer (Agilent Technologies, Santa Clara, CA). Each sample was labeled using the Affymetrix IVT Express Kit according to the manufacturer's protocol. The GeneChip Human Genome U133A 2.0 (Affymterix, Santa Clara, CA), representing 14,500 well‐characterized genes, was hybridized with the labeled RNA using GeneChip Hybridization Wash and Stain Kit (#702232) in the Affymetrix GeneChip Hybridization Oven 640, according to the manufacturer's instructions. Following hybridization, the arrays were washed and stained using the Affymetrix GeneChip Fluidics Station 450 according to the manufacturer's protocol and scanned using the GeneChip Scanner 3000 7G. The scanned image file (DAT) and the intensity data (CEL) was imported into ArrayStar (DNASTAR, Inc., Madison, WI). The Robust Multi‐array Average (RMA) was used to normalize the data. The nine slides were grouped based on treatment and Student *t*‐test with asymptotic *P*‐value and Benjamini–Hochberg multiple corrections was performed comparing CE versus DMSO and 90MX versus DMSO. At a *P*‐value of 0.05 and a twofold change, a total of 236 entities were significantly regulated by CE while there were no genes that met the criteria for 90MX. The group of genes was examined by hierarchical clustering using complete linkage analysis of the normalized data (JMP 7.0; SAS Institute, Cary, NC). Gene Ontology (GO) and pathway analysis was performed, using Ingenuity Pathway Analysis (Qiagen, Redwood City, CA).

### Statistical analysis

One‐way analysis of variance, followed by Dunnett's post hoc test, was used to test the difference between treatments (*P* < 0.05). The values were expressed as mean ± SEM. All data analyses were performed by JMP 7.0 (SAS Institute) and data plotted by Prism 5.01 (GraphPad Software, Inc., San Diego, CA).

## Results

### Regulation of inflammation‐related genes in THP‐1 cells

**Figure 1:**
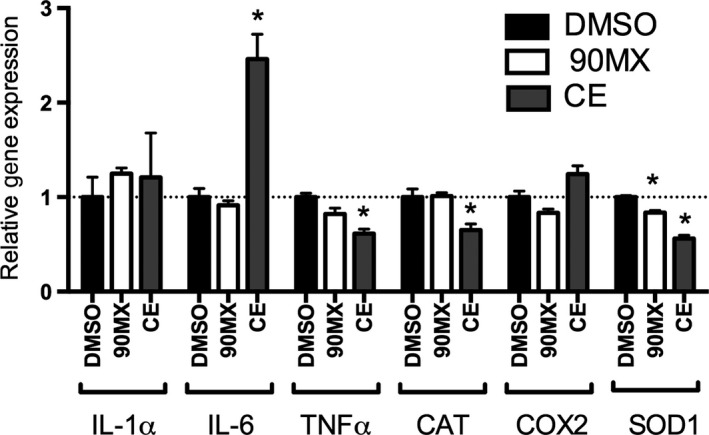
Regulation of inflammation‐related genes by cranberry extracts in THP‐1 cells. Cells were treated and RNA extracted as described in Materials and Methods. Gene expression following treatment with DMSO (0.1% v/v),CE or 90MX (100 *μ*g/mL) is expressed relative to a housekeeping gene (18S) and normalized to vehicle control (DMSO). Asterisks denote significantly different than control (*P* < 0.05, *n* = 3, bars represent mean and SEM).

The ability of CEs to regulate proinflammatory gene expression in THP‐1 cells was examined using an LPS‐challenge model (Lee et al. 2009; Zhang et al. 2010). A single concentration of each extract (100 *μ*g/mL) was examined; there was no sign of overt toxicity at this dose (data not shown). The 90MX powder had no effect on expression of interleukin‐1*α* (IL‐1*α*), interleukin‐6 (IL6), tumor necrosis factor‐*α* (TNF*α*), catalase (CAT), cyclooxygenase‐2 (COX2) or superoxide dismutase 1 (SOD1) mRNA (Fig. 1). The CE extract at this dose resulted in a significant increase in IL‐6 mRNA (2.5‐fold) with decreased expression of TNF*α*, CAT and SOD1 mRNA (1.6, 1.5 and 1.8‐fold, respectively) relative to the vehicle control. The pattern of gene expression, with several LPS‐induced genes being repressed while others are either unaffected or augmented, suggests an atypical mechanism of action and prompted a more comprehensive examination of mRNA concentration.

### Comprehensive analysis of gene expression

**Figure 2:**
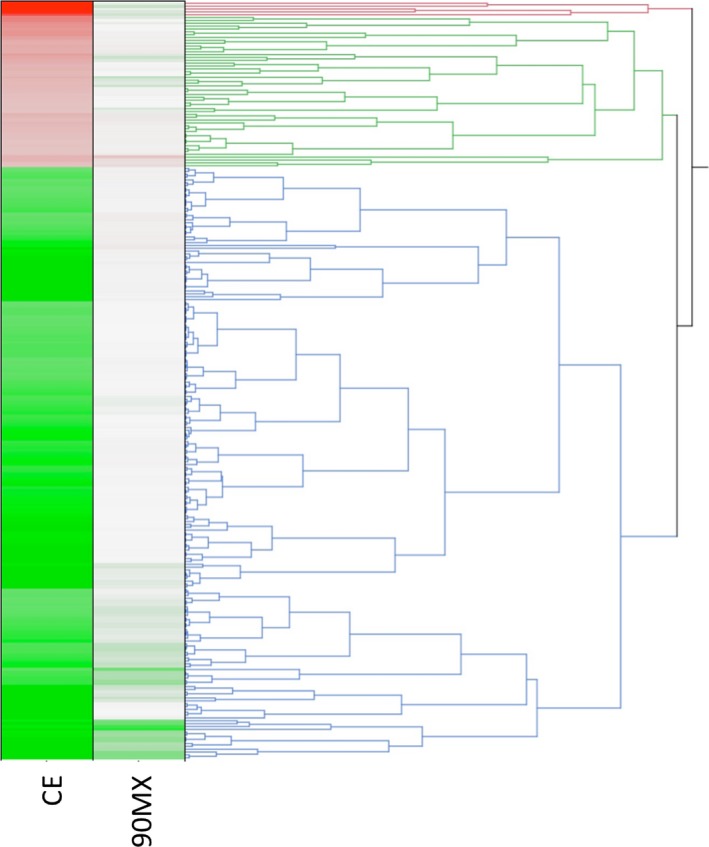
Comprehensive analysis of gene expression. Analysis of gene expression by high‐density microarrays was performed by microarrays as described in Material and Methods. Average expression values (*n* = 3 arrays per group) were exported to JMP (SAS Institute, Cary NC) and hierarchical clustering performed. Data were expressed relative to the DMSO‐treated group with green representing a decrease and red an increase relative to control (depicted as white).

The design of the present experiments was aimed to understand the anti‐inflammatory effects of cranberry polyphenol extracts to identify sensitive biomarkers for subsequent studies and to begin understanding their potential mechanism of action. The THP‐1 monocytes were treated with CE or 90MX at 100 *μ*g/mL for 16 h, followed by stimulation with LPS and the RNA was used to examine gene expression by high‐density microarray. A total of 236 genes were significantly regulated by CE with a twofold change (Table S1 and Fig. 2) with 64 genes reaching a threefold threshold (Table 3). There were no significant changes using these same criteria with the 90MX extract. A majority of the genes (78%) were repressed by CE; Metallothionein 1F (MT1F) mRNA was increased fivefold while interferon‐induced protein with tetratricopeptide repeats 5 (IFIT5) was reduced 10‐fold relative to the DMSO‐treated group.

**Table 3:** Genes significantly regulated by cranberry extracts (threefold, *P* < 0.05)

Probe set ID	Gene symbol	Gene title	90MX	CE
213629_x_at	MT1F	Metallothionein 1F	0.80	4.84
211906_s_at	SERPINB4	Serpin peptidase inhibitor, clade B (ovalbumin), member 4	0.70	4.47
210524_x_at	MT1F	Metallothionein 1F	0.81	4.10
212859_x_at	MT1E	Metallothionein 1E	0.75	3.87
220322_at	IL36G	Interleukin 36, gamma	0.72	3.56
203213_at	CDK1	Cyclin‐dependent kinase 1	0.89	0.33
210764_s_at	CYR61	Cysteine‐rich, angiogenic inducer, 61	0.87	0.33
203725_at	GADD45A	Growth arrest and DNA‐damage‐inducible, alpha	0.94	0.32
207386_at	CYP7B1	Cytochrome P450, family 7, subfamily B, polypeptide 1	1.05	0.32
211668_s_at	PLAU	Plasminogen activator, urokinase	1.04	0.32
218662_s_at	NCAPG	Non‐SMC condensin I complex, subunit G	0.91	0.32
214453_s_at	IFI44	Interferon‐induced protein 44	0.57	0.31
204747_at	IFIT3	Interferon‐induced protein with tetratricopeptide repeats 3	0.63	0.31
203362_s_at	MAD2L1	MAD2 mitotic arrest deficient‐like 1 (yeast)	0.94	0.31
219209_at	IFIH1	Interferon induced with helicase C domain 1	0.80	0.31
202127_at	PRPF4B	Pre‐mRNA processing factor 4B	0.56	0.30
201291_s_at	TOP2A	Topoisomerase (DNA) II alpha 170 kDa	0.96	0.30
218883_s_at	CENPU	Centromere protein U	0.74	0.30
213226_at	CCNA2	Cyclin A2	0.80	0.29
202508_s_at	SNAP25	Synaptosomal‐associated protein, 25 kDa	0.51	0.29
209969_s_at	STAT1	Signal transducer and activator of transcription 1, 91 kDa	0.80	0.29
210559_s_at	CDK1	Cyclin‐dependent kinase 1	0.90	0.29
211597_s_at	HOPX	HOP homeobox	0.99	0.29
216442_x_at	FN1	Fibronectin 1	1.00	0.28
206513_at	AIM2	Absent in melanoma 2	0.85	0.28
203967_at	CDC6	Cell division cycle 6	0.77	0.28
204127_at	RFC3	Replication factor C (activator 1) 3, 38 kDa	0.81	0.28
204994_at	MX2	Myxovirus (influenza virus) resistance 2 (mouse)	0.63	0.28
210495_x_at	FN1	Fibronectin 1	0.98	0.28
212464_s_at	FN1	Fibronectin 1	0.95	0.27
203708_at	PDE4B	Phosphodiesterase 4B, cAMP‐specific	0.78	0.27
211719_x_at	FN1	Fibronectin 1	0.98	0.27
202833_s_at	SERPINA1	Serpin peptidase inhibitor, clade A (alpha‐1 antiproteinase, antitrypsin), member 1	1.03	0.27
205479_s_at	PLAU	Plasminogen activator, urokinase	1.02	0.27
201506_at	TGFBI	Transforming growth factor, beta‐induced, 68 kDa	1.12	0.26
204823_at	NAV3	Neuron navigator 3	0.94	0.26
220104_at	ZC3HAV1	Zinc finger CCCH‐type, antiviral 1	0.79	0.26
213293_s_at	TRIM22	Tripartite motif containing 22	0.41	0.26
222162_s_at	ADAMTS1	ADAM metallopeptidase with thrombospondin type 1 motif, 1	0.90	0.26
201340_s_at	ENC1	Ectodermal‐neural cortex 1 (with BTB domain)	0.99	0.25
201341_at	ENC1	Ectodermal‐neural cortex 1 (with BTB domain)	0.93	0.25
202628_s_at	SERPINE1	Serpin peptidase inhibitor, clade E (nexin, plasminogen activator inhibitor type 1), member 1	0.99	0.24
219148_at	PBK	PDZ‐binding kinase	0.99	0.24
202086_at	MX1	Myxovirus (influenza virus) resistance 1, interferon‐inducible protein p78 (mouse)	0.70	0.23
218039_at	NUSAP1	Nucleolar and spindle‐associated protein 1	0.85	0.23
201890_at	RRM2	Ribonucleotide reductase M2	0.89	0.22
202589_at	TYMS	Thymidylate synthetase	0.85	0.21
219691_at	SAMD9	Sterile alpha motif domain containing 9	0.62	0.21
205552_s_at	OAS1	2′‐5′‐oligoadenylate synthetase 1, 40/46 kDa	0.54	0.20
202627_s_at	SERPINE1	Serpin peptidase inhibitor, clade E (nexin, plasminogen activator inhibitor type 1), member 1	1.01	0.20
214146_s_at	PPBP	Proplatelet basic protein (chemokine (C‐X‐C motif) ligand 7)	1.26	0.20
219684_at	RTP4	Receptor (chemosensory) transporter protein 4	0.72	0.20
219908_at	DKK2	Dickkopf WNT signaling pathway inhibitor 2	0.88	0.19
218585_s_at	DTL	Denticleless E3 ubiquitin protein ligase homolog (Drosophila)	0.95	0.19
203596_s_at	IFIT5	Interferon‐induced protein with tetratricopeptide repeats 5	0.77	0.19
213294_at	EIF2AK2	Eukaryotic translation initiation factor 2‐alpha kinase 2	0.75	0.18
209773_s_at	RRM2	Ribonucleotide reductase M2	0.86	0.18
202869_at	OAS1	2′‐5′‐oligoadenylate synthetase 1, 40/46 kDa	0.54	0.16
205239_at	AREG	Amphiregulin	1.00	0.15
212977_at	ACKR3	Atypical chemokine receptor 3	1.06	0.15
203153_at	IFIT1	Interferon‐induced protein with tetratricopeptide repeats 1	0.54	0.14
201289_at	CYR61	Cysteine‐rich, angiogenic inducer, 61	0.83	0.13
218943_s_at	DDX58	DEAD (Asp‐Glu‐Ala‐Asp) box polypeptide 58	0.69	0.11
203595_s_at	IFIT5	Interferon‐induced protein with tetratricopeptide repeats 5	0.75	0.09

**Figure 3:**
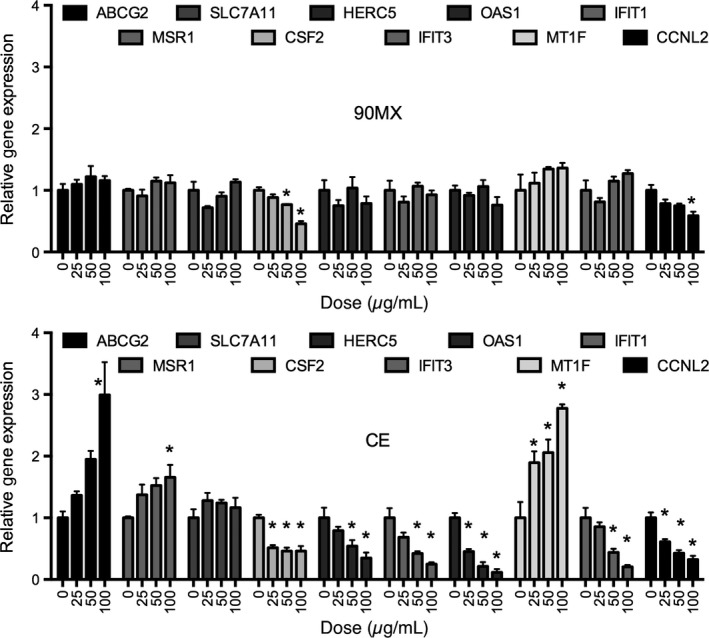
Expression of selected genes identified as responsive to cranberry extracts in THP‐1 cells. Genes were selected from Table 2 to Figure 2 for validation by real‐time PCR. Cells were treated and RNA extracted as described in Material and Methods. Gene expression following treatment with 90MX or CE (0, 25, 50, 100 *μ*g/mL) is expressed relative to a housekeeping gene (18S) and normalized to vehicle control (DMSO). Asterisks denote significantly different than control (*P* < 0.05, *n* = 3, bars represent mean and SEM).

Quantitative real‐time PCR was used to confirm a subset of transcripts identified by the microarray experiments. Care was taken to choose genes that were both increased and decreased by CE treatment as well as those with known biological functions. THP‐1 cells were treated as described above, with the exception that three doses of each extract was examined. As shown in Figure 3 (top panel), unlike the microarray experiment, the 90MX extract significantly regulated colony‐stimulating factor 2 (CSF2) and cyclin L2 (CCNL2) mRNA accumulation. All genes studied, with the exception of solute carrier family 7A11 (SLC7A11), were significantly regulated by CE, albeit to a different extent and with varying potency (Fig. 3, bottom panel). However, in the ATP‐binding cassette, the sub‐family G, member 2 (ABCG2) mRNA was affected only at the highest dose, CSF2, 2′‐5′‐oligoadenylate synthetase 1 (OAS1), metallothionein 1F (MT1F), and CCNL2 mRNA were regulated at the 25 *μ*g/mL dose.

### GO and pathway analysis

The list of genes that were significantly regulated by the CE extract in THP‐1 cells (twofold and *P* < 0.05, 236 genes) were classified according to their functional ontology terms (GO). Several GO biological processes were significantly enriched in these CE‐regulated genes (http://pantherdb.org, see Table S2), including immune effector process, response to interferon, cell cycle checkpoint, cell cycle phase, and response to metal ion. The gene expression patterns were further examined to determine if they were consistent with certain diseases and functions (Ingenuity Pathway Analysis; Table 4 and Figures S1, S2). Genes regulated by CEs, such as ABCG2, CCNA2, CCNB1, and CDC25A were associated with cell cycle control and DNA repair processes. The majority of the genes in this network were decreased in expression relative to the DMSO‐treated cells. Similarly, several CE‐regulated genes were enriched in the inflammatory response network and most genes, with the exception of IL6, were repressed. Potential mechanisms by which CE regulates genes within these networks is shown in Table 5. Several transcription factors and signaling molecules are implicated, including HGF signaling, PPAR, LXR, or VDR activation and importantly inhibition of inflammatory signaling such as interferon, MAPK, or NFAT activity.

**Table 4:** Gene ontology analysis: top diseases and functions

ID	Top diseases and functions	Molecules in network
1	Cell cycle, cellular assembly and organization, DNA replication, recombination, and repair	ABCG2, alcohol group acceptor phosphotransferase, ALT, ASAH1, AURKA, BUB1, BUB1B, CCNA2, CCNB1, CD163, Cdc2, CDC25A, CDK1, Cyclin B, ENO2, GADD45A, GINS1, HISTONE, Histone H1, Histone h3, HOPX, IL6, MCM4, MT1G, NASP, NCAPG, PRC1, PRPF4B, PTPase, RNA polymerase II, SFPQ, SMC4, SNX10, Sod, TTK
2	Cell cycle, embryonic development, organismal survival	AIM2, APC (complex), ATM/ATR, ATR, CDC6, CDC20, Cdk, Cyclin A, Cyclin D, Cyclin E, DTL, Dynein, E2f, FANCI, GMNN, HERC5, LUC7L3, MAD2L1, MCM10, MELK, NBN, NFkB (complex), NUSAP1, PCNA, PPP4R4, Rb, Rfc, RFC3, RFC4, RPA, RRM2, SKP2, TRIM22, TYMS, UNG
3	Antimicrobial response, inflammatory response, cell signaling	2′ 5′ oas, DDX58, DDX60, EIF2AK2, ERK, IFI16, IFI44, IFIH1, IFIT1, IFIT3, IFIT5, Ifn, IFN alpha/beta, IFN Beta, IFN type 1, Ifnar, IRF, ISG15, ISG20, ISGF3, JAK, MT1M, MX1, Oas, OAS1, OAS2, PARP12, RSAD2, STAT4, Stat1‐Stat2, THEMIS2, USP18, XAF1, ZC3HAV1
4	Nervous system development and function, cancer, organismal injury and abnormalities	ACKR3, ACPP, ADAMTS1, ADCY, ADRB, Alpha catenin, AREG, BNIP3, CAP2, Cg, Creb, DUSP2, EMR2, FGF13, FSH, Gpcr, GPR56, GPR65, Hdac, HRH1, Lh, Mapk, Metalloprotease, MT1F, MT1X, NMDA Receptor, OXTR, Pdgfr, PLC, PVR, SLC7A11, SNAP25, STEAP1, TCF, Vegf
5	Infectious disease, cell morphology, hair and skin development, and function	AIF1, Akt, c‐Src, Calcineurin A, Collagen(s), CYR61, Fascin, FCGR2B, FERMT1, Fgf, Fibrin, Fibrinogen, GDF15, Integrin, Integrin alpha 3 beta 1, Integrin alpha 4 beta 1, ITGA1, JINK1/2, KIAA0101, Laminin, Lfa‐1, LRIG1, MAP2K1/2, MT1E, Notch, PBK, PMAIP1, PNISR, PNN, PPIG, SLC4A7, TLR2/TLR4, TRIB3, TRIM5, ZWINT

**Table 5:** Gene ontology analysis: top pathways

Canonical pathways	*z*‐score	Molecules
Cell cycle: G2/M DNA damage checkpoint regulation	1.134	GADD45A, TOP2A, ATR, AURKA, CDK1, SKP2, CCNB1
HGF signaling	1	MET, FOS, IL6, ELK3, PRKCB
PPAR signaling	1	IL33, FOS, IL36G, IL1A
LXR/RXR activation	0.447	IL33, IL36G, IL1A, SERPINA1, IL6
Activation of IRF by cytosolic pattern recognition receptors	−0.447	IFIH1, IL10, DDX58, IL6, STAT1, ISG15
PDGF signaling	−0.447	FOS, CAV1, EIF2AK2, STAT1, PRKCB
IL‐6 signaling	−0.447	IL33, FOS, IL36G, IL1A, IL6
NF‐*κ*B signaling	−0.447	IL33, IL36G, IL1A, EIF2AK2, PRKCB
ERK/MAPK signaling	−0.447	FOS, STAT1, ELK3, DUSP2, PRKCB
Cholecystokinin/gastrin‐mediated signaling	−0.816	IL33, FOS, IL36G, IL1A, MEF2C, PRKCB
Toll‐like receptor signaling	−1	IL33, FOS, IL36G, IL1A, EIF2AK2
Growth hormone signaling	−1	FOS, IGFBP3, STAT1, PRKCB
JAK/stat signaling	−1	STAT4, FOS, IL6, STAT1
TREM1 signaling	−1	IL10, IL6, FCGR2B, CSF2
Tec kinase signaling	−1	STAT4, FOS, STAT1, PRKCB
Production of nitric oxide and reactive oxygen species in macrophages	−1	FOS, SERPINA1, STAT1, PRKCB
Colorectal cancer metastasis signaling	−1	FOS, IL6, STAT1, MMP19
Role of CHK proteins in cell cycle checkpoint control	−1.342	PCNA, RFC4, ATR, CDK1, CDC25A, RFC3, NBN
HMGB1 signaling	−1.342	FOS, IL1A, IL6, SERPINE1, CSF2, PLAT
p38 MAPK signaling	−1.342	IL33, IL36G, IL1A, MEF2C, STAT1
Aryl hydrocarbon receptor signaling	−1.342	CCNA2, FOS, IL1A, ATR, IL6
Acute phase response signaling	−1.414	IL33, FOS, IL36G, IL1A, FN1, SERPINA1, IL6, SERPINE1
VDR/RXR activation	−2	GADD45A, IGFBP3, CYP27B1, CSF2, PRKCB
UVA‐induced MAPK signaling	−2	FOS, ZC3HAV1, PARP12, STAT1
Role of NFAT in regulation of the immune response	−2	FOS, MEF2C, FCGR2B, RCAN2
Interferon signaling	−2.236	IFIT3, IFIT1, OAS1, MX1, STAT1

## Discussion

Several berry fruits, including the American cranberry (*Vaccinium macrocarpon*), have recently received attention as a result of their effects in vitro and their associations with observational studies with lowered risk of some chronic diseases (Zafra‐Stone et al. 2007; Blumberg et al. 2013). Cranberries, consumed in sauces, juices, dried fruit, or taken as a dietary supplement in powders or extract form, are a rich source of polyphenols that are associated with a variety of biological effects. In vitro studies have demonstrated antibacterial, antiviral, antimutagenic, anticarcinogenic, antitumorigenic, antiangiogenic, anti‐inflammatory, and antioxidant properties (Yan et al. 2002; Bagchi et al. 2004; Ferguson et al. 2004; Seeram et al. 2004; Neto 2007; Zafra‐Stone et al. 2007) (3,7,8). In vivo, animal models reveal that CEs: reduce proinflammatory interleukins (Kim et al. 2011); suppress Helicobacter pylori infection (Blumberg et al. 2013), and; improve pancreatic *β*‐cell glucose responsiveness and *β*‐cell mass (Zhu et al. 2011). The clinical effects of cranberry and their bioactives include: lowering LDL cholesterol and total cholesterol (Basu and Lyons 2012); improving markers of endothelial function (Bagchi et al. 2004; Dohadwala et al. 2011), lowering glycemic responses (Wilson et al. 2010), elevating plasma antioxidant capacity (McKay et al. 2015); modulation of gastric *Helicobacter pylori* infection (Shmuely et al. 2012); reducing biomarkers of metabolic syndrome (Wilson et al. 2010; Basu and Lyons 2012; Simão et al. 2013), and; protecting against urinary tract infections (UTIs) (Foxman et al. 2015).

Cranberries are a particularly rich source of phenolic phytochemicals, including phenolic acids (benzoic, hydroxycinnamic, and ellagic acids) and flavonoids (anthocyanins, flavonols, and flavanols), and these bioactive molecules are believed to be associated with the health benefits described above. The major anthocyanins in cranberry are galactosides and arabinosides of cyanidin and peonidin (Neto 2007). Anthocyanin content varies widely among cranberry cultivars with averages between 25 and 65 mg per 100 g of ripe fruit at harvest. The primary flavonol in cranberries is quercetin, which exists in several glycosidic forms, and the total flavonol content of cranberry fruit typically in the range of 20–30 mg per 100 g of fresh fruit weight. In comparing the two CEs used in the present study (Martín et al. 2015), 90MX contained higher amount of organic acids while CE had higher quantities of highly bioactive compounds such as phenolic acids, flavonols, and flavanols (see Table 1). More specific, components with a high antioxidant capacity such as chlorogenic acid, epicatechin, and quercetin were found in high concentrations in CE. The total PACs concentration was 18 times higher in CE than 90MX (51,000 vs. 2800 mg/100 g; [Martín et al. 2015]). In HepG2 cells, both CEs decreased ROSs and protected the cells from t‐BOOH‐induced cytotoxicity, albeit with CE being much more potent. Interestingly, stress‐related signaling through the JNK pathway by t‐BOOH was not averted by CE, indicating another mechanism such as redox regulation, might be primary in the protection from oxidative stress by CE in HepG2 cells.

The CEs significantly decreased expression of TNF*α*, CAT, and SOD1 mRNA (Fig. 1) relative to the vehicle control in LPS‐stimulated THP‐1 cells. In addition, the microarray studies showed mRNA for acute phase response genes (IL33, FOS, IL36G, IL1A, FN1, SERPINA1, SERPINE1) and interferon‐signaling genes (IFIT3, IFIT1, OAS1, MX1, STAT1; Table 4) were decreased in these cells. These responses are consistent with several other studies showing an anti‐oxidant and anti‐inflammatory effect of cranberries and their bioactive molecules, in particular the PACs. In rats fed an atherogenic diet, C‐reactive protein (CRP) and IL‐1*β* were significantly lower in the cranberry powder groups compared to those in control rats (Kim et al. 2011). A CE (OptiBerry) significantly inhibited basal MCP‐1 and inducible NF‐*κ*B transcription as well as the inflammatory biomarker IL‐8 in a similar in vivo model (Zafra‐Stone et al. 2007). Treatment of Caco‐2 cells with these CEs prevented iron/ascorbate‐mediated lipid peroxidation and counteracted LPS‐mediated inflammation as evidenced by the decrease in proinflammatory cytokines (TNF‐*α* and IL‐6), cyclo‐oxygenase‐2 (COX‐2) and prostaglandin E2 (PGE2, [Denis et al. 2015]). Networks of genes involved in the antimicrobial and inflammatory responses and infectious disease (Table 3) were identified as being coordinately affected by CE, adding support for the associations seen in observational studies. The moderate increase in IL‐6 mRNA observed in the present studies, in the absence of increases in other proinflammatory markers such as COX‐2 and IL‐1*α*, may be the result of several factors. First, this suggests that CE is not causing generalized oxidative stress since other mRNAs would be coordinately affected. Second, this increase in IL6 mRNA may not be indicative of altered activity due to the small increase observed (2.5‐fold), the fact that protein level of this factor was not examined and IL6‐responsive genes (such as IL1) were not altered. Finally, it is obvious that CE, due to the complex mixture of bioactive molecules, has multiple mechanisms of action.

The mechanism of this anti‐inflammatory effect has not been extensively studied, although PACs from cranberries and other sources have been proposed to act through oxygen free radical scavenging (Li et al. 2001) as well as metal chelation (Arola‐Arnal and Bladé 2011), a contributor to ROS production. PACs affect the activity of signaling molecules associated with the inflammatory response, including the redox‐sensitive transcription factor NF‐*κ*B (Liu et al. 2014), 5′‐AMP activated protein kinase (AMPK)(Cui et al. 2012), p38 mitogen‐activated protein kinase (MAPK, [Dinh et al. 2014]) and JNK (Guha et al. 2013; Liu et al. 2014). Data presented herein support the premise that CE decreases activity of NF‐*κ*B, p38 MAPK and NFAT in LPS‐challenged macrophages. Quercetin, a polyphenol found in high concentration in CE (400 mg/100 g; [Martín et al. 2015]), induces metallothionein (MT) expression by activating the phosphorylation of JNK, p38 and PI3K/Akt as well as by enhancing Nrf2 DNA‐binding activity in HepG2 cells (Weng et al. 2011). In CE‐treated THP‐1 cells, MT1E and 1F mRNA were among the most robustly induced transcripts, indicating that similar signaling pathways are affected in this cell line and that CEs may protect against metal‐induced oxidative stress and toxicity.

Several in vitro studies suggest that cranberry bioactives are potential therapeutic agents for the treatment of cancer (Neto 2007; Zafra‐Stone et al. 2007). For example, CEs and PACs decrease proliferation of the human breast (Ferguson et al. 2004; Sun and Liu 2006), glioblastoma multiforme (U87), colon (HT‐29), prostate (DU145), and oral cancer cell lines (Seeram et al. 2004; Ferguson et al. 2006). Cell cycle arrest and apoptosis was induced by a cranberry PAC‐rich extract in human esophageal adenocarcinoma (EAC) cells (Kresty et al. 2008) and prostate cells (MacLean et al. 2011). Although cell cycle and proliferation was not studied herein, several genes involved in checkpoint control such as PCNA, RFC4, ATR, CDK1, CDC25A, RFC3, and NBN (Tables 3, 5) were induced at the mRNA level by CE in THP‐1 cells. Interestingly, cranberry ethanolic extract (CEE) prevents the DNA damage produced by benzo[a]pyrene (B[a]P) in an in vivo mouse peripheral blood micronucleus assay (Madrigal‐Santillán et al. 2012). Our current study showed that several CE inducible genes are involved in G2/M DNA Damage checkpoint regulation, including GADD45A and CCNB1 (Tables 4, 5).

Taken together, these studies support the role of cranberry and its complex and rich phytochemical composition as an adjuvant for the treatment or prevention of chronic diseases with an inflammatory component. CEs decreased expression of several markers of inflammation and ROS signaling in the human monocyte cell line THP‐1 that had been challenged with LPS. In addition, through the use of comprehensive gene expression analysis, the potential for cranberry or extracts for the prevention of metal‐induced toxicity as well as cancer is supported. A limitation of the studies presented is that they are exclusively in vitro, using surrogate model systems for inflammation and oxidative stress. Although predictive of the potential of CE to affect human health, there must be consideration of availability of the bioactive constituents and the concentration achieved in vivo. In support of an in vivo response and hence bioavailability of active constituents, rats and mice‐fed CEs exhibited reduced CRP, proinflammatory interleukins, and increase NO synthesis (Wang et al. 2012; Blumberg et al. 2013; Foxman et al. 2015). The range of doses of the cranberry phenolic powders was selected according to previous studies showing that concentrations in the *μ*g/mL range with 30–40 *μ*mol/L of cranberry phytochemicals have been found in plasma after cranberry juice intake (Martín et al. 2015). Although the identity of the specific cranberry‐derived bioactive molecule or the molecular pathway involved has not been definitively identified, this study supports a wide range of in vitro, animal as well as nutritional invention studies showing this berry have unique and powerful health benefits.

## Conflict of Interest

JVH is an employee of Penn State University and has a financial stake with Indigo Biosciences Inc., which may constitute a conflict of interest.

## Supporting information


**Table S1.** Genes significantly regulated by CE (*P* < 0.05, twofold).
**Table S2.** PANTHER over‐representation test.
**Figure S1.** Cell proliferation network affected by CE. Genes significantly affected by CE were examined by Ingenuity Pathway Analysis (Qiagen). Red filled symbols denotes increased, while green filled symbols denote decreased, expression relative to control (DMSO‐treated cells).
**Figure S2.** Inflammation network affected by CE. See legend to Figure S1.
