# Supplementary material for: Effects of cranberry extracts on gene expression in THP‐1 cells
Source: Food Sci Nutr. 2016 Apr 25;5(1):148–59. doi: 10.1002/fsn3.374 (PMC5217924; doi:10.1002/fsn3.374)
Supplement: Supplementary file 1 — Table S1. Genes significantly regulated by CE (P < 0.05, twofold). Table S2. PANTHER over‐representation test. Figure S1. Cell proliferation network affected by CE. Genes significantly affected by CE were examined by Ingenuity Pathway Analysis (Qiagen). Red filled symbols denotes increased, while green filled symbols denote decreased, expression relative to control (DMSO‐treated cells). Figure S2. Inflammation network affected by CE. See legend to Figure S1. [file FSN3-5-148-s001.docx]

SUPPLEMENTAL DATA

Effects of cranberry extracts on gene expression in THP-1 cells.

Daniel B. Hannon^1^, Jerry T. Thompson^1^, Christina Khoo^2^ , Vijaya Juturu^3 ,^ and John P. Vanden Heuvel ^1,4,*^

**Abbreviated Title:** Effect of cranberry extracts on Gene Expression

***Corresponding Author** Jack Vanden Heuvel, PhD, Professor of Molecular Toxicology, Penn State University, 325 Life Sciences Building, University Park, PA 16802, T: (814) 863-8532, F: (814) 863-1696, [jpv2@psu.edu](mailto:jpv2@psu.edu)

| **Supplemental Table 1. Genes significantly regulated by CE (p<0.05, 2-fold)** | | | | |
| --- | --- | --- | --- | --- |
| **Probe Set ID** | **Gene Symbol** | **Gene Title** | **CE** | **90MX** |
| 213629_x_at | MT1F | metallothionein 1F | 4.837 | 0.797 |
| 211906_s_at | SERPINB4 | serpin peptidase inhibitor, clade B (ovalbumin), member 4 | 4.469 | 0.697 |
| 210524_x_at | |  | 4.105 | 0.814 |
| 212859_x_at | MT1E | metallothionein 1E | 3.872 | 0.755 |
| 220322_at | IL36G | interleukin 36, gamma | 3.557 | 0.715 |
| 201849_at | BNIP3 | BCL2/adenovirus E1B 19kDa interacting protein 3 | 2.963 | 0.767 |
| 214660_at | ITGA1 | integrin, alpha 1 | 2.921 | 0.861 |
| 217165_x_at | MT1F | metallothionein 1F | 2.750 | 0.801 |
| 205207_at | IL6 | interleukin 6 | 2.729 | 1.059 |
| 219915_s_at | SLC16A10 | solute carrier family 16 (aromatic amino acid transporter), member 10 | 2.723 | 0.920 |
| 218237_s_at | SLC38A1 | solute carrier family 38, member 1 | 2.699 | 1.079 |
| 205110_s_at | FGF13 | fibroblast growth factor 13 | 2.642 | 0.895 |
| 217546_at | MT1M | metallothionein 1M | 2.566 | 0.778 |
| 221577_x_at | GDF15 | growth differentiation factor 15 | 2.532 | 1.108 |
| 210413_x_at | SERPINB3, SERPINB4 | serpin peptidase inhibitor, clade B (ovalbumin), member 3; serpin peptidase inhibitor, clade B (ovalbumin), member 4 | 2.454 | 0.710 |
| 210991_s_at | RIMS3 | regulating synaptic membrane exocytosis 3 | 2.423 | 1.073 |
| 221523_s_at | RRAGD | Ras-related GTP binding D | 2.418 | 1.173 |
| 218404_at | SNX10 | sorting nexin 10 | 2.383 | 1.142 |
| 204745_x_at | MT1G | metallothionein 1G | 2.369 | 0.778 |
| 209735_at | ABCG2 | ATP-binding cassette, sub-family G (WHITE), member 2 | 2.350 | 1.213 |
| 201942_s_at | CPD | carboxypeptidase D | 2.345 | 1.093 |
| 206239_s_at | SPINK1 | serine peptidase inhibitor, Kazal type 1 | 2.329 | 0.937 |
| 202620_s_at | PLOD2 | procollagen-lysine, 2-oxoglutarate 5-dioxygenase 2 | 2.289 | 0.677 |
| 204326_x_at | MT1X | metallothionein 1X | 2.277 | 0.834 |
| 215435_at |  |  | 2.259 | 1.943 |
| 216109_at | MED13L | mediator complex subunit 13-like | 2.257 | 1.543 |
| 203498_at | RCAN2 | regulator of calcineurin 2 | 2.250 | 0.965 |
| 207528_s_at | SLC7A11 | solute carrier family 7 (anionic amino acid transporter light chain, xc- system), member 11 | 2.246 | 1.283 |
| 209685_s_at | PRKCB | protein kinase C, beta | 2.217 | 1.166 |
| 217678_at | SLC7A11 | solute carrier family 7 (anionic amino acid transporter light chain, xc- system), member 11 | 2.206 | 1.280 |
| 208581_x_at | MT1X | metallothionein 1X | 2.205 | 0.751 |
| 212554_at | CAP2 | CAP, adenylate cyclase-associated protein, 2 (yeast) | 2.196 | 0.901 |
| 201848_s_at | BNIP3 | BCL2/adenovirus E1B 19kDa interacting protein 3 | 2.195 | 0.697 |
| 215723_s_at | PLD1 | phospholipase D1, phosphatidylcholine-specific | 2.194 | 0.873 |
| 206461_x_at | MT1H | metallothionein 1H | 2.184 | 0.787 |
| 202073_at | OPTN | optineurin | 2.167 | 1.117 |
| 201313_at | ENO2 | enolase 2 (gamma, neuronal) | 2.165 | 1.016 |
| 35820_at | GM2A | GM2 ganglioside activator | 2.155 | 1.096 |
| 210367_s_at | PTGES | prostaglandin E synthase | 2.146 | 1.025 |
| 221524_s_at | RRAGD | Ras-related GTP binding D | 2.134 | 1.129 |
| 220898_at |  |  | 2.129 | 1.270 |
| 201943_s_at | CPD | carboxypeptidase D | 2.105 | 0.928 |
| 215761_at | DMXL2 | Dmx-like 2 | 2.101 | 1.457 |
| 220882_at |  |  | 2.077 | 1.097 |
| 203510_at | MET | MET proto-oncogene, receptor tyrosine kinase | 2.075 | 1.089 |
| 212737_at | GM2A | GM2 ganglioside activator | 2.070 | 1.021 |
| 201968_s_at | PGM1 | phosphoglucomutase 1 | 2.059 | 0.951 |
| 221899_at | N4BP2L2 | NEDD4 binding protein 2-like 2 | 2.053 | 1.567 |
| 202619_s_at | PLOD2 | procollagen-lysine, 2-oxoglutarate 5-dioxygenase 2 | 2.045 | 0.751 |
| 219410_at | TMEM45A | transmembrane protein 45A | 2.040 | 0.881 |
| 218149_s_at | ZNF395 | zinc finger protein 395 | 2.039 | 0.909 |
| 218088_s_at | RRAGC | Ras-related GTP binding C | 2.035 | 1.059 |
| 213902_at | ASAH1 | N-acylsphingosine amidohydrolase (acid ceramidase) 1 | 2.033 | 1.187 |
| 219270_at | CHAC1 | ChaC, cation transport regulator homolog 1 (E. coli) | 2.025 | 1.123 |
| 212907_at | SLC30A1 | solute carrier family 30 (zinc transporter), member 1 | 2.020 | 1.114 |
| 216336_x_at | MT1E | metallothionein 1E | 2.009 | 0.804 |
| 215891_s_at | GM2A | GM2 ganglioside activator | 2.006 | 1.092 |
| 218145_at | TRIB3 | tribbles pseudokinase 3 | 2.001 | 1.072 |
| 205579_at | HRH1 | histamine receptor H1 | 0.500 | 1.071 |
| 221919_at | HNRNPA1 | heterogeneous nuclear ribonucleoprotein A1 | 0.499 | 0.831 |
| 212662_at | PVR | poliovirus receptor | 0.498 | 1.031 |
| 211429_s_at | SERPINA1 | serpin peptidase inhibitor, clade A (alpha-1 antiproteinase, antitrypsin), member 1 | 0.498 | 1.148 |
| 203968_s_at | CDC6 | cell division cycle 6 | 0.498 | 0.800 |
| 60474_at | FERMT1 | fermitin family member 1 | 0.496 | 0.911 |
| 219918_s_at | ASPM | asp (abnormal spindle) homolog, microcephaly associated (Drosophila) | 0.495 | 0.905 |
| 214314_s_at | EIF5B | eukaryotic translation initiation factor 5B | 0.495 | 0.737 |
| 210118_s_at | IL1A | interleukin 1, alpha | 0.494 | 0.886 |
| 208079_s_at | AURKA | aurora kinase A | 0.494 | 0.976 |
| 204794_at | DUSP2 | dual specificity phosphatase 2 | 0.494 | 0.997 |
| 206825_at | OXTR | oxytocin receptor | 0.492 | 0.895 |
| 210889_s_at | FCGR2B | Fc fragment of IgG, low affinity IIb, receptor (CD32) | 0.492 | 1.000 |
| 212621_at | TMEM194A | transmembrane protein 194A | 0.492 | 0.938 |
| 212179_at | PNISR | PNN-interacting serine/arginine-rich protein | 0.491 | 0.769 |
| 209901_x_at | AIF1 | allograft inflammatory factor 1 | 0.490 | 0.977 |
| 202888_s_at | ANPEP | alanyl (membrane) aminopeptidase | 0.490 | 1.094 |
| 220651_s_at | MCM10 | minichromosome maintenance complex component 10 | 0.490 | 0.869 |
| 210052_s_at | TPX2 | TPX2, microtubule-associated | 0.490 | 0.965 |
| 208389_s_at | SLC1A2 | solute carrier family 1 (glial high affinity glutamate transporter), member 2 | 0.489 | 0.895 |
| 212847_at | FUBP1 | far upstream element (FUSE) binding protein 1 | 0.488 | 0.838 |
| 201008_s_at | TXNIP | thioredoxin interacting protein | 0.487 | 1.155 |
| 219012_s_at | C11orf30 | chromosome 11 open reading frame 30 | 0.485 | 0.779 |
| 219211_at | USP18 | ubiquitin specific peptidase 18 | 0.484 | 0.729 |
| 214093_s_at | FUBP1 | far upstream element (FUSE) binding protein 1 | 0.484 | 0.723 |
| 204803_s_at | RRAD | Ras-related associated with diabetes | 0.484 | 1.021 |
| 212037_at | PNN | pinin, desmosome associated protein | 0.484 | 0.963 |
| 209189_at | FOS | FBJ murine osteosarcoma viral oncogene homolog | 0.484 | 1.061 |
| 212070_at | GPR56 | G protein-coupled receptor 56 | 0.483 | 1.122 |
| 218796_at | FERMT1 | fermitin family member 1 | 0.482 | 0.930 |
| 202906_s_at | NBN | nibrin | 0.482 | 0.868 |
| 203485_at | RTN1 | reticulon 1 | 0.482 | 1.176 |
| 207433_at | IL10 | interleukin 10 | 0.481 | 0.868 |
| 217544_at | MIR3916 | microRNA 3916 | 0.481 | 0.507 |
| 202363_at | SPOCK1 | sparc/osteonectin, cwcv and kazal-like domains proteoglycan (testican) 1 | 0.480 | 1.012 |
| 207703_at | NLGN4Y | neuroligin 4, Y-linked | 0.480 | 1.043 |
| 213007_at | FANCI | Fanconi anemia, complementation group I | 0.480 | 0.818 |
| 219825_at | CYP26B1 | cytochrome P450, family 26, subfamily B, polypeptide 1 | 0.480 | 0.905 |
| 221773_at | ELK3 | ELK3, ETS-domain protein (SRF accessory protein 2) | 0.478 | 0.938 |
| 205992_s_at | IL15 | interleukin 15 | 0.477 | 0.741 |
| 208965_s_at | IFI16 | interferon, gamma-inducible protein 16 | 0.476 | 0.974 |
| 209684_at | RIN2 | Ras and Rab interactor 2 | 0.476 | 0.858 |
| 202330_s_at | UNG | uracil-DNA glycosylase | 0.475 | 0.910 |
| 217299_s_at | NBN | nibrin | 0.474 | 0.913 |
| 209903_s_at | ATR | ATR serine/threonine kinase | 0.473 | 0.873 |
| 203625_x_at | SKP2 | S-phase kinase-associated protein 2, E3 ubiquitin protein ligase | 0.472 | 0.993 |
| 210286_s_at | SLC4A7 | solute carrier family 4, sodium bicarbonate cotransporter, member 7 | 0.469 | 1.104 |
| 207610_s_at | EMR2 | egf-like module containing, mucin-like, hormone receptor-like 2 | 0.469 | 1.086 |
| 204279_at | PSMB9 | proteasome (prosome, macropain) subunit, beta type, 9 | 0.468 | 0.877 |
| 201970_s_at | NASP | nuclear autoantigenic sperm protein (histone-binding) | 0.468 | 0.867 |
| 206336_at | CXCL6 | chemokine (C-X-C motif) ligand 6 | 0.467 | 0.997 |
| 204575_s_at | MMP19 | matrix metallopeptidase 19 | 0.466 | 0.894 |
| 207165_at | HMMR | hyaluronan-mediated motility receptor (RHAMM) | 0.465 | 0.996 |
| 204802_at | RRAD | Ras-related associated with diabetes | 0.465 | 1.097 |
| 202430_s_at | PLSCR1 | phospholipid scramblase 1 | 0.464 | 0.680 |
| 212097_at | CAV1 | caveolin 1, caveolae protein, 22kDa | 0.464 | 0.883 |
| 204023_at | RFC4 | replication factor C (activator 1) 4, 37kDa | 0.463 | 0.855 |
| 218543_s_at | PARP12 | poly (ADP-ribose) polymerase family, member 12 | 0.462 | 0.772 |
| 209642_at | BUB1 | BUB1 mitotic checkpoint serine/threonine kinase | 0.462 | 0.927 |
| 208771_s_at | LOC101928830, LTA4H | uncharacterized LOC101928830, leukotriene A4 hydrolase | 0.461 | 1.071 |
| 212030_at | RBM25 | RNA binding motif protein 25 | 0.461 | 0.853 |
| 211596_s_at | LRIG1 | leucine-rich repeats and immunoglobulin-like domains 1 | 0.460 | 0.884 |
| 203804_s_at | LUC7L3 | LUC7-like 3 (S. cerevisiae) | 0.456 | 0.703 |
| 205483_s_at | ISG15 | ISG15 ubiquitin-like modifier | 0.455 | 0.679 |
| 207375_s_at | IL15RA | interleukin 15 receptor, alpha | 0.455 | 0.830 |
| 213051_at | ZC3HAV1 | zinc finger CCCH-type, antiviral 1 | 0.453 | 0.901 |
| 210229_s_at | CSF2 | colony stimulating factor 2 (granulocyte-macrophage) | 0.453 | 0.809 |
| 219494_at | RAD54B | RAD54 homolog B (S. cerevisiae) | 0.453 | 0.749 |
| 204698_at | ISG20 | interferon stimulated exonuclease gene 20kDa | 0.452 | 0.672 |
| 203645_s_at | CD163 | CD163 molecule | 0.452 | 1.122 |
| 212060_at | U2SURP | U2 snRNP-associated SURP domain containing | 0.452 | 0.910 |
| 208982_at | PECAM1 | platelet/endothelial cell adhesion molecule 1 | 0.451 | 1.116 |
| 208995_s_at | PPIG | peptidylprolyl isomerase G (cyclophilin G) | 0.451 | 0.773 |
| 220672_at | PPP4R4 | protein phosphatase 4, regulatory subunit 4 | 0.450 | 0.979 |
| 210705_s_at | TRIM5 | tripartite motif containing 5 | 0.450 | 0.635 |
| 218067_s_at | ARGLU1 | arginine and glutamate rich 1 | 0.446 | 0.637 |
| 205226_at | PDGFRL | platelet-derived growth factor receptor-like | 0.445 | 0.804 |
| 218757_s_at | UPF3B | UPF3 regulator of nonsense transcripts homolog B (yeast) | 0.444 | 0.753 |
| 209198_s_at | SYT11 | synaptotagmin XI | 0.441 | 0.995 |
| 214016_s_at | SFPQ | splicing factor proline/glutamine-rich | 0.439 | 0.708 |
| 204462_s_at | SLC16A2 | solute carrier family 16, member 2 (thyroid hormone transporter) | 0.438 | 0.861 |
| 207571_x_at | THEMIS2 | thymocyte selection associated family member 2 | 0.438 | 0.929 |
| 222037_at | MCM4 | minichromosome maintenance complex component 4 | 0.438 | 0.806 |
| 201202_at | PCNA | proliferating cell nuclear antigen | 0.436 | 0.782 |
| 209761_s_at | SP110 | SP110 nuclear body protein | 0.433 | 0.869 |
| 204521_at | FAM216A | family with sequence similarity 216, member A | 0.433 | 1.013 |
| 219352_at | HERC6 | HECT and RLD domain containing E3 ubiquitin protein ligase family member 6 | 0.431 | 0.593 |
| 212176_at | PNISR | PNN-interacting serine/arginine-rich protein | 0.430 | 0.632 |
| 215049_x_at | CD163 | CD163 molecule | 0.428 | 1.163 |
| 221427_s_at | CCNL2 | cyclin L2 | 0.428 | 0.579 |
| 204211_x_at | EIF2AK2 | eukaryotic translation initiation factor 2-alpha kinase 2 | 0.427 | 0.837 |
| 209902_at | ATR | ATR serine/threonine kinase | 0.427 | 0.929 |
| 218350_s_at | GMNN | geminin, DNA replication inhibitor | 0.427 | 0.777 |
| 209200_at | MEF2C | myocyte enhancer factor 2C | 0.423 | 0.963 |
| 206118_at | STAT4 | signal transducer and activator of transcription 4 | 0.423 | 0.733 |
| 38149_at | ARHGAP25 | Rho GTPase activating protein 25 | 0.423 | 0.783 |
| 204882_at | ARHGAP25 | Rho GTPase activating protein 25 | 0.422 | 0.836 |
| 210785_s_at | THEMIS2 | thymocyte selection associated family member 2 | 0.422 | 0.947 |
| 204825_at | MELK | maternal embryonic leucine zipper kinase | 0.421 | 0.869 |
| 219471_at | KIAA0226L | KIAA0226-like | 0.421 | 1.006 |
| 205569_at | LAMP3 | lysosomal-associated membrane protein 3 | 0.419 | 0.793 |
| 206632_s_at | APOBEC3B | apolipoprotein B mRNA editing enzyme, catalytic polypeptide-like 3B | 0.416 | 1.034 |
| 201860_s_at | PLAT | plasminogen activator, tissue | 0.415 | 1.127 |
| 201663_s_at | SMC4 | structural maintenance of chromosomes 4 | 0.412 | 0.820 |
| 206102_at | GINS1 | GINS complex subunit 1 (Psf1 homolog) | 0.411 | 0.917 |
| 44790_s_at | KIAA0226L | KIAA0226-like | 0.411 | 1.013 |
| 203755_at | BUB1B | BUB1 mitotic checkpoint serine/threonine kinase B | 0.411 | 0.860 |
| 212036_s_at | PNN | pinin, desmosome associated protein | 0.410 | 0.713 |
| 204393_s_at | ACPP | acid phosphatase, prostate | 0.402 | 1.036 |
| 214710_s_at | CCNB1 | cyclin B1 | 0.400 | 0.952 |
| 204695_at | CDC25A | cell division cycle 25A | 0.396 | 0.947 |
| 211581_x_at | LST1 | leukocyte specific transcript 1 | 0.393 | 0.914 |
| 203214_x_at | CDK1 | cyclin-dependent kinase 1 | 0.393 | 0.904 |
| 206133_at | XAF1 | XIAP associated factor 1 | 0.392 | 0.767 |
| 218986_s_at | DDX60 | DEAD (Asp-Glu-Ala-Asp) box polypeptide 60 | 0.390 | 0.682 |
| 218782_s_at | ATAD2 | ATPase family, AAA domain containing 2 | 0.389 | 0.849 |
| 202786_at | STK39 | serine threonine kinase 39 | 0.388 | 0.931 |
| 219978_s_at | NUSAP1 | nucleolar and spindle associated protein 1 | 0.383 | 1.005 |
| 211582_x_at | LST1 | leukocyte specific transcript 1 | 0.378 | 0.946 |
| 209959_at | NR4A3 | nuclear receptor subfamily 4, group A, member 3 | 0.376 | 0.907 |
| 214574_x_at | LST1 | leukocyte specific transcript 1 | 0.376 | 0.923 |
| 202748_at | GBP2 | guanylate binding protein 2, interferon-inducible | 0.376 | 0.971 |
| 214974_x_at | CXCL5 | chemokine (C-X-C motif) ligand 5 | 0.375 | 1.144 |
| 200644_at | MARCKSL1 | MARCKS-like 1 | 0.375 | 1.031 |
| 219863_at | HERC5 | HECT and RLD domain containing E3 ubiquitin protein ligase 5 | 0.375 | 0.524 |
| 205542_at | STEAP1 | six transmembrane epithelial antigen of the prostate 1 | 0.374 | 1.004 |
| 214467_at | GPR65 | G protein-coupled receptor 65 | 0.374 | 1.251 |
| 202446_s_at | PLSCR1 | phospholipid scramblase 1 | 0.373 | 0.704 |
| 218009_s_at | PRC1 | protein regulator of cytokinesis 1 | 0.371 | 0.872 |
| 204026_s_at | ZWINT | ZW10 interacting kinetochore protein | 0.368 | 0.907 |
| 215633_x_at | LST1 | leukocyte specific transcript 1 | 0.368 | 0.991 |
| 207978_s_at | NR4A3 | nuclear receptor subfamily 4, group A, member 3 | 0.366 | 0.862 |
| 209821_at | IL33 | interleukin 33 | 0.365 | 0.856 |
| 210629_x_at | LST1 | leukocyte specific transcript 1 | 0.365 | 0.946 |
| 205676_at | CYP27B1 | cytochrome P450, family 27, subfamily B, polypeptide 1 | 0.365 | 0.913 |
| 204285_s_at | PMAIP1 | phorbol-12-myristate-13-acetate-induced protein 1 | 0.364 | 0.847 |
| 217317_s_at | HERC2P2, HERC2P9 | hect domain and RLD 2 pseudogene 2, hect domain and RLD 2 pseudogene 9 | 0.359 | 0.439 |
| 203760_s_at | SLA | Src-like-adaptor | 0.358 | 0.964 |
| 201010_s_at | TXNIP | thioredoxin interacting protein | 0.352 | 1.066 |
| 214181_x_at | LST1 | leukocyte specific transcript 1 | 0.351 | 0.967 |
| 213797_at | RSAD2 | radical S-adenosyl methionine domain containing 2 | 0.350 | 0.590 |
| 204286_s_at | PMAIP1 | phorbol-12-myristate-13-acetate-induced protein 1 | 0.348 | 0.897 |
| 202503_s_at | KIAA0101 | KIAA0101 | 0.347 | 0.918 |
| 210095_s_at | IGFBP3 | insulin-like growth factor binding protein 3 | 0.343 | 0.917 |
| 204822_at | TTK | TTK protein kinase | 0.343 | 0.758 |
| 204972_at | OAS2 | 2'-5'-oligoadenylate synthetase 2, 69/71kDa | 0.341 | 0.602 |
| 202870_s_at | CDC20 | cell division cycle 20 | 0.341 | 1.041 |
| 212143_s_at | IGFBP3 | insulin-like growth factor binding protein 3 | 0.339 | 0.948 |
| 203761_at | SLA | Src-like-adaptor | 0.337 | 0.947 |
| 203213_at | CDK1 | cyclin-dependent kinase 1 | 0.332 | 0.887 |
| 210764_s_at | CYR61 | cysteine-rich, angiogenic inducer, 61 | 0.332 | 0.867 |
| 203725_at | GADD45A | growth arrest and DNA-damage-inducible, alpha | 0.323 | 0.940 |
| 207386_at | CYP7B1 | cytochrome P450, family 7, subfamily B, polypeptide 1 | 0.318 | 1.055 |
| 211668_s_at | PLAU | plasminogen activator, urokinase | 0.318 | 1.040 |
| 218662_s_at | NCAPG | non-SMC condensin I complex, subunit G | 0.315 | 0.910 |
| 214453_s_at | IFI44 | interferon-induced protein 44 | 0.313 | 0.567 |
| 204747_at | IFIT3 | interferon-induced protein with tetratricopeptide repeats 3 | 0.312 | 0.634 |
| 203362_s_at | MAD2L1 | MAD2 mitotic arrest deficient-like 1 (yeast) | 0.310 | 0.938 |
| 219209_at | IFIH1 | interferon induced with helicase C domain 1 | 0.306 | 0.795 |
| 202127_at | PRPF4B | pre-mRNA processing factor 4B | 0.305 | 0.565 |
| 201291_s_at | TOP2A | topoisomerase (DNA) II alpha 170kDa | 0.304 | 0.957 |
| 218883_s_at | CENPU | centromere protein U | 0.301 | 0.735 |
| 213226_at | CCNA2 | cyclin A2 | 0.293 | 0.796 |
| 202508_s_at | SNAP25 | synaptosomal-associated protein, 25kDa | 0.291 | 0.506 |
| 209969_s_at | STAT1 | signal transducer and activator of transcription 1, 91kDa | 0.290 | 0.802 |
| 210559_s_at | CDK1 | cyclin-dependent kinase 1 | 0.289 | 0.900 |
| 211597_s_at | HOPX | HOP homeobox | 0.289 | 0.989 |
| 216442_x_at | FN1 | fibronectin 1 | 0.285 | 0.999 |
| 206513_at | AIM2 | absent in melanoma 2 | 0.284 | 0.848 |
| 203967_at | CDC6 | cell division cycle 6 | 0.284 | 0.767 |
| 204127_at | RFC3 | replication factor C (activator 1) 3, 38kDa | 0.281 | 0.805 |
| 204994_at | MX2 | myxovirus (influenza virus) resistance 2 (mouse) | 0.280 | 0.631 |
| 210495_x_at | FN1 | fibronectin 1 | 0.278 | 0.975 |
| 212464_s_at | FN1 | fibronectin 1 | 0.271 | 0.946 |
| 203708_at | PDE4B | phosphodiesterase 4B, cAMP-specific | 0.268 | 0.777 |
| 211719_x_at | FN1 | fibronectin 1 | 0.266 | 0.981 |
| 202833_s_at | SERPINA1 | serpin peptidase inhibitor, clade A (alpha-1 antiproteinase, antitrypsin), member 1 | 0.265 | 1.030 |
| 205479_s_at | PLAU | plasminogen activator, urokinase | 0.265 | 1.024 |
| 201506_at | TGFBI | transforming growth factor, beta-induced, 68kDa | 0.263 | 1.122 |
| 204823_at | NAV3 | neuron navigator 3 | 0.262 | 0.941 |
| 220104_at | ZC3HAV1 | zinc finger CCCH-type, antiviral 1 | 0.262 | 0.792 |
| 213293_s_at | TRIM22 | tripartite motif containing 22 | 0.261 | 0.407 |
| 222162_s_at | ADAMTS1 | ADAM metallopeptidase with thrombospondin type 1 motif, 1 | 0.260 | 0.901 |
| 201340_s_at | ENC1 | ectodermal-neural cortex 1 (with BTB domain) | 0.254 | 0.987 |
| 201341_at | ENC1 | ectodermal-neural cortex 1 (with BTB domain) | 0.246 | 0.927 |
| 202628_s_at | SERPINE1 | serpin peptidase inhibitor, clade E (nexin, plasminogen activator inhibitor type 1), member 1 | 0.244 | 0.985 |
| 219148_at | PBK | PDZ binding kinase | 0.242 | 0.987 |
| 202086_at | MX1 | myxovirus (influenza virus) resistance 1, interferon-inducible protein p78 (mouse) | 0.228 | 0.697 |
| 218039_at | NUSAP1 | nucleolar and spindle associated protein 1 | 0.227 | 0.849 |
| 201890_at | RRM2 | ribonucleotide reductase M2 | 0.216 | 0.889 |
| 202589_at | TYMS | thymidylate synthetase | 0.210 | 0.851 |
| 219691_at | SAMD9 | sterile alpha motif domain containing 9 | 0.208 | 0.621 |
| 205552_s_at | OAS1 | 2'-5'-oligoadenylate synthetase 1, 40/46kDa | 0.205 | 0.543 |
| 202627_s_at | SERPINE1 | serpin peptidase inhibitor, clade E (nexin, plasminogen activator inhibitor type 1), member 1 | 0.202 | 1.011 |
| 214146_s_at | PPBP | pro-platelet basic protein (chemokine (C-X-C motif) ligand 7) | 0.200 | 1.256 |
| 219684_at | RTP4 | receptor (chemosensory) transporter protein 4 | 0.199 | 0.723 |
| 219908_at | DKK2 | dickkopf WNT signaling pathway inhibitor 2 | 0.189 | 0.878 |
| 218585_s_at | DTL | denticleless E3 ubiquitin protein ligase homolog (Drosophila) | 0.188 | 0.951 |
| 203596_s_at | IFIT5 | interferon-induced protein with tetratricopeptide repeats 5 | 0.185 | 0.767 |
| 213294_at | EIF2AK2 | eukaryotic translation initiation factor 2-alpha kinase 2 | 0.181 | 0.750 |
| 209773_s_at | RRM2 | ribonucleotide reductase M2 | 0.181 | 0.859 |
| 202869_at | OAS1 | 2'-5'-oligoadenylate synthetase 1, 40/46kDa | 0.164 | 0.543 |
| 205239_at | AREG | amphiregulin | 0.152 | 0.997 |
| 212977_at | ACKR3 | atypical chemokine receptor 3 | 0.146 | 1.064 |
| 203153_at | IFIT1 | interferon-induced protein with tetratricopeptide repeats 1 | 0.137 | 0.544 |
| 201289_at | CYR61 | cysteine-rich, angiogenic inducer, 61 | 0.128 | 0.827 |
| 218943_s_at | DDX58 | DEAD (Asp-Glu-Ala-Asp) box polypeptide 58 | 0.108 | 0.690 |
| 203595_s_at | IFIT5 | interferon-induced protein with tetratricopeptide repeats 5 | 0.091 | 0.754 |

| **Supplemental Table 2. PANTHER Overrepresentation Test** | | | | | |
| --- | --- | --- | --- | --- | --- |
| **GO biological process complete** | **Reference** | **Found** | **Expected** | **Enrichment** | **P-value** |
| defense response to virus | 155 | 25 | 1.56 | > 5 | 1.86E-18 |
| response to biotic stimulus | 705 | 43 | 7.08 | > 5 | 1.61E-17 |
| response to other organism | 676 | 42 | 6.79 | > 5 | 2.58E-17 |
| response to external biotic stimulus | 676 | 42 | 6.79 | > 5 | 2.58E-17 |
| defense response to other organism | 365 | 32 | 3.67 | > 5 | 1.28E-16 |
| response to virus | 235 | 27 | 2.36 | > 5 | 1.93E-16 |
| response to cytokine | 620 | 37 | 6.23 | > 5 | 2.89E-14 |
| cytokine-mediated signaling pathway | 388 | 26 | 3.9 | > 5 | 3.10E-10 |
| type I interferon signaling pathway | 68 | 13 | 0.68 | > 5 | 3.23E-09 |
| cellular response to type I interferon | 68 | 13 | 0.68 | > 5 | 3.23E-09 |
| response to type I interferon | 69 | 13 | 0.69 | > 5 | 3.87E-09 |
| **immune effector process** | **437** | **26** | **4.39** | **> 5** | **4.48E-09** |
| cellular response to cytokine stimulus | 519 | 28 | 5.21 | > 5 | 5.49E-09 |
| **cell cycle checkpoint** | **258** | **20** | **2.59** | **> 5** | **2.42E-08** |
| mitotic cell cycle phase | 294 | 20 | 2.95 | > 5 | 2.41E-07 |
| **cell cycle phase** | **296** | **20** | **2.97** | **> 5** | **2.71E-07** |
| regulation of multi-organism process | 448 | 24 | 4.5 | > 5 | 3.12E-07 |
| **response to metal ion** | **265** | **19** | **2.66** | **> 5** | **3.20E-07** |
| biological phase | 300 | 20 | 3.01 | > 5 | 3.43E-07 |
| negative regulation of viral genome replication | 45 | 10 | 0.45 | > 5 | 4.04E-07 |
| response to inorganic substance | 378 | 22 | 3.8 | > 5 | 4.56E-07 |
| negative regulation of viral process | 86 | 12 | 0.86 | > 5 | 9.34E-07 |
| regulation of viral genome replication | 68 | 11 | 0.68 | > 5 | 1.21E-06 |
| negative regulation of viral life cycle | 83 | 11 | 0.83 | > 5 | 9.53E-06 |
| negative regulation of multi-organism process | 146 | 13 | 1.47 | > 5 | 3.37E-05 |
| DNA replication | 211 | 15 | 2.12 | > 5 | 4.28E-05 |
| response to cadmium ion | 37 | 8 | 0.37 | > 5 | 4.35E-05 |
| cellular response to zinc ion | 15 | 6 | 0.15 | > 5 | 1.01E-04 |
| regulation of symbiosis, encompassing mutualism through parasitism | 209 | 14 | 2.1 | > 5 | 2.89E-04 |
| response to lipopolysaccharide | 252 | 15 | 2.53 | > 5 | 4.28E-04 |
| mitotic nuclear division | 333 | 17 | 3.34 | > 5 | 4.88E-04 |
| regulation of viral process | 185 | 13 | 1.86 | > 5 | 5.20E-04 |
| mitotic prometaphase | 99 | 10 | 0.99 | > 5 | 6.72E-04 |
| response to molecule of bacterial origin | 266 | 15 | 2.67 | > 5 | 8.53E-04 |
| mitotic cell cycle phase transition | 306 | 16 | 3.07 | > 5 | 8.77E-04 |
| cell cycle phase transition | 310 | 16 | 3.11 | > 5 | 1.04E-03 |
| regulation of proteasomal ubiquitin-dependent protein catabolic process | 136 | 11 | 1.37 | > 5 | 1.37E-03 |
| response to transition metal nanoparticle | 108 | 10 | 1.08 | > 5 | 1.48E-03 |
| cellular response to metal ion | 109 | 10 | 1.09 | > 5 | 1.61E-03 |
| regulation of proteolysis involved in cellular protein catabolic process | 280 | 15 | 2.81 | > 5 | 1.63E-03 |
| regulation of viral life cycle | 173 | 12 | 1.74 | > 5 | 1.92E-03 |
| regulation of cellular protein catabolic process | 296 | 15 | 2.97 | > 5 | 3.26E-03 |
| cellular response to cadmium ion | 15 | 5 | 0.15 | > 5 | 4.14E-03 |
| cellular response to inorganic substance | 123 | 10 | 1.24 | > 5 | 4.79E-03 |
| chromosome condensation | 30 | 6 | 0.3 | > 5 | 5.72E-03 |
| response to interferon-beta | 18 | 5 | 0.18 | > 5 | 1.01E-02 |
| mitotic cell cycle checkpoint | 169 | 11 | 1.7 | > 5 | 1.13E-02 |
| cell cycle G1/S phase transition | 172 | 11 | 1.73 | > 5 | 1.33E-02 |
| G1/S transition of mitotic cell cycle | 172 | 11 | 1.73 | > 5 | 1.33E-02 |
| anaphase-promoting complex-dependent proteasomal ubiquitin-dependent protein catabolic process | 83 | 8 | 0.83 | > 5 | 1.88E-02 |
| regulation of proteasomal protein catabolic process | 217 | 12 | 2.18 | > 5 | 1.99E-02 |
| mitotic M phase | 221 | 12 | 2.22 | > 5 | 2.39E-02 |
| M phase | 223 | 12 | 2.24 | > 5 | 2.61E-02 |
| mitotic S phase | 117 | 9 | 1.17 | > 5 | 2.74E-02 |
| regulation of ubiquitin-protein transferase activity | 118 | 9 | 1.18 | > 5 | 2.93E-02 |
| acute-phase response | 40 | 6 | 0.4 | > 5 | 2.96E-02 |
| S phase | 119 | 9 | 1.19 | > 5 | 3.13E-02 |
| regulation of ubiquitin-protein ligase activity involved in mitotic cell cycle | 90 | 8 | 0.9 | > 5 | 3.39E-02 |
| regulation of DNA replication | 122 | 9 | 1.23 | > 5 | 3.82E-02 |
| response to zinc ion | 42 | 6 | 0.42 | > 5 | 3.90E-02 |
| positive regulation of response to external stimulus | 233 | 12 | 2.34 | > 5 | 4.06E-02 |
| regulation of ligase activity | 124 | 9 | 1.25 | > 5 | 4.35E-02 |
| acute inflammatory response | 66 | 7 | 0.66 | > 5 | 4.38E-02 |
| cellular response to extracellular stimulus | 197 | 11 | 1.98 | > 5 | 4.77E-02 |
| regulation of chromosome segregation | 67 | 7 | 0.67 | > 5 | 4.82E-02 |


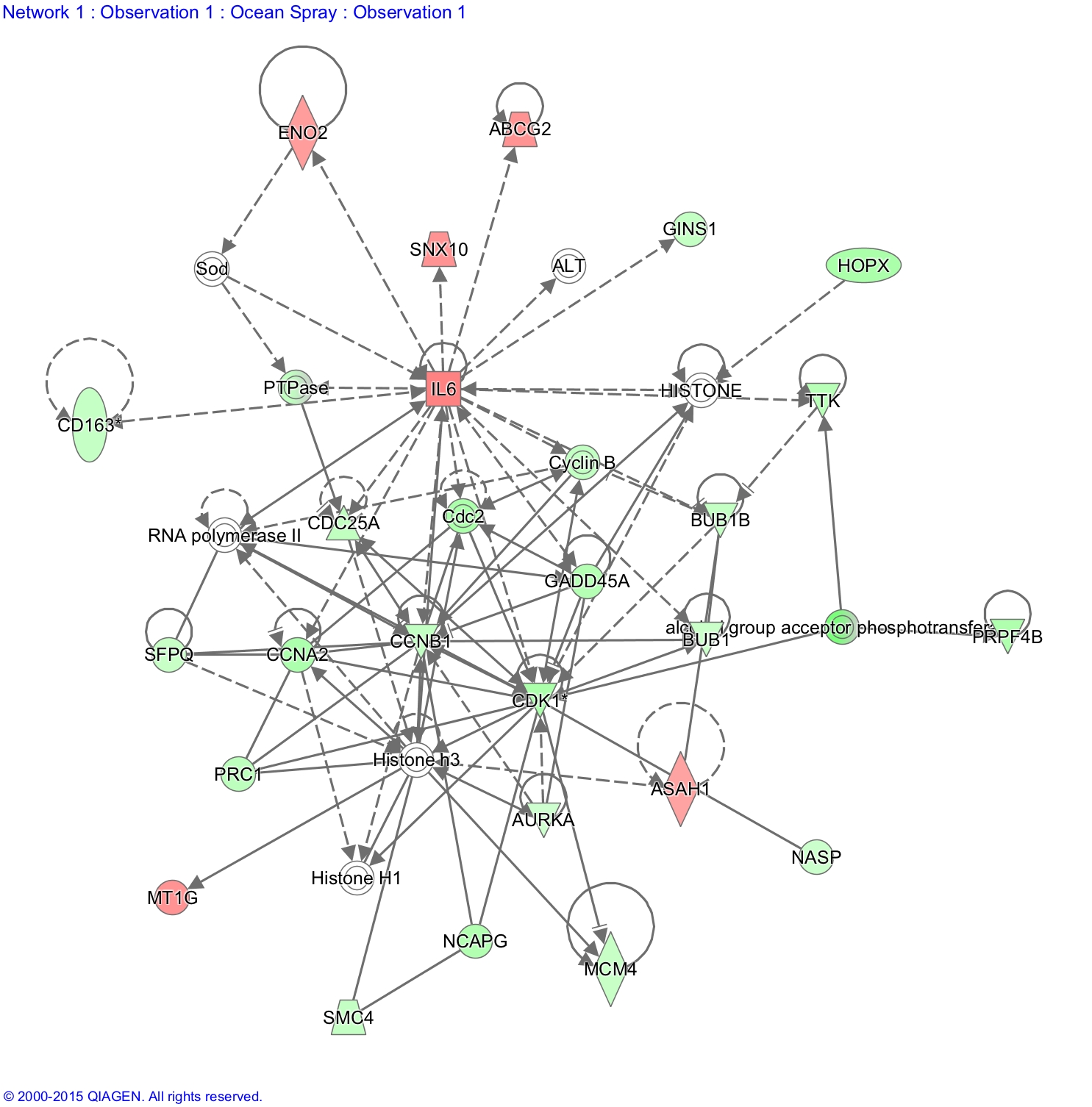


Supplemental Figure 1. Cell proliferation network affected by CE. Genes significantly affected by CE were examined by Ingenuity Pathway Analysis (Qiagen, Redwood City, CA). Red filled symbols denotes increased, while green filled symbols denote decreased, expression relative to control (DMSO treated cells).


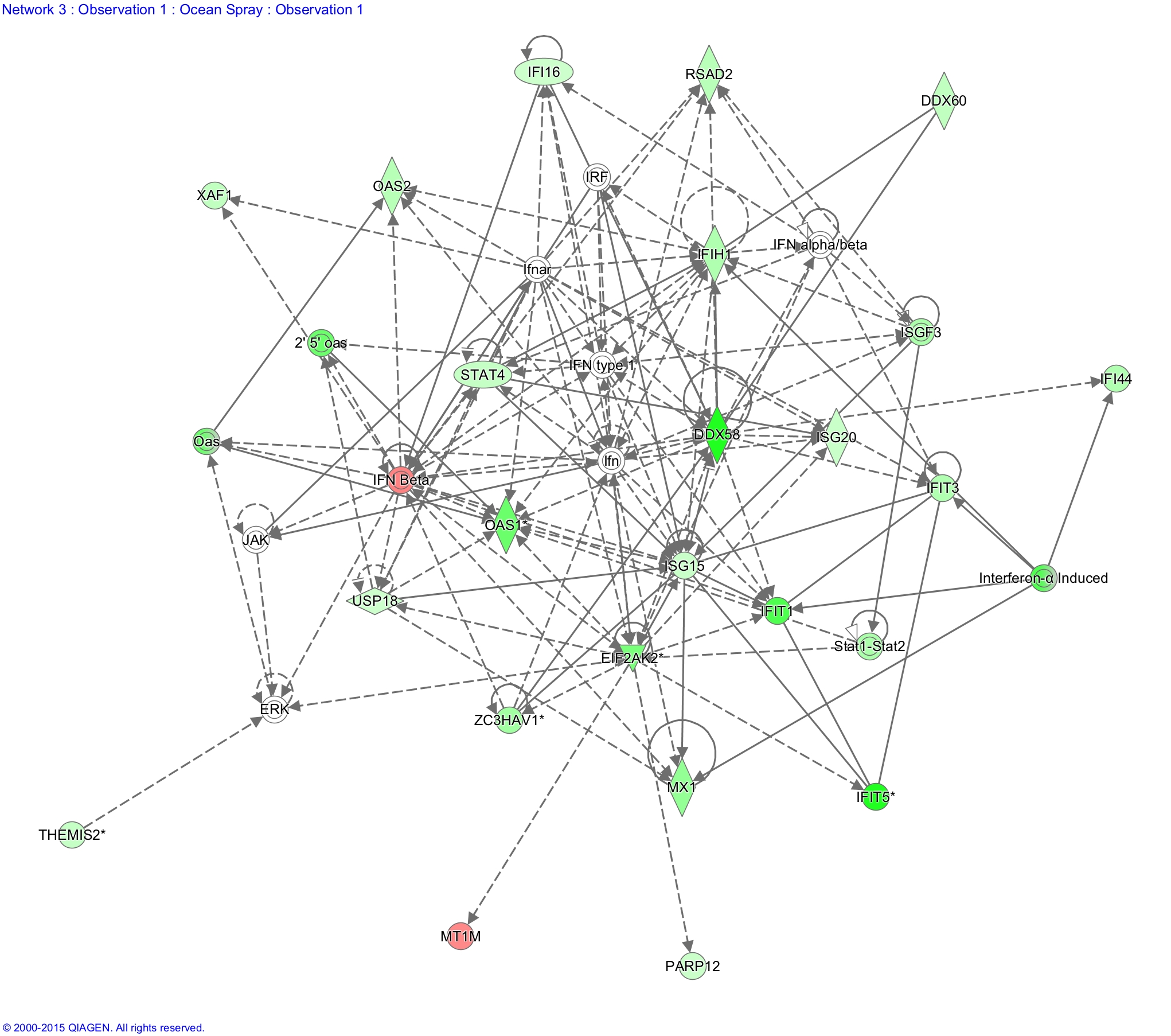


Supplemental Figure 2. Inflammation network affected by CE. See legend to Supplemental Figure 1.
